# Effects of Seven Sterilization Methods on the Functional Characteristics and Color of Yan 73 (*Vitis vinifera*) Grape Juice

**DOI:** 10.3390/foods12203722

**Published:** 2023-10-10

**Authors:** Zixian Zhao, Jiaqi Wang, Caihong Li, Yuanke Zhang, Xiangyu Sun, Tingting Ma, Qian Ge

**Affiliations:** 1 Quality Standards and Testing Institute of Agricultural Technology, Yinchuan 750002, China; 2022051208@nwsuaf.edu.cn (Z.Z.); lch.6868@163.com (C.L.); 2 College of Enology, Viti-Viniculture Engineering Technology Center of State Forestry and Grassland Administration, Shaanxi Engineering Research Center for Viti-Viniculture, Heyang Viti-Viniculture Station, Northwest A&F University, Yangling 712100, China; hello-wjq@nwafu.edu.cn (J.W.); zhangyk2001@nwafu.edu.cn (Y.Z.); sunxiangyu@nwafu.edu.cn (X.S.); 3 College of Food Science and Engineering, Northwest A&F University, Yangling 712100, China

**Keywords:** Yan73 grape juice, thermal sterilization, non-thermal inactivation, functional indicators, color

## Abstract

Yan 73 (*Vitis vinifera*) is a dyed grape variety cultivated in China. Currently, most studies have focused on the mechanism of anthocyanins or the impact of anthocyanins as auxiliary color varieties on wine color. There is little research on its direct use or direct processing of products such as juice. In order to investigate the effects of different processing methods on the juice of Yan 73 grapes, the physicochemical and functional properties, as well as the sensory indexes of the juice, were analyzed by using thermal pasteurization (TP), thermosonication (TS), TS combined with nisin (TSN), TS combined with ε-Polylysine (TSε), irradiation (IR), and high hydrostatic pressure (HHP). The physicochemical indexes, functional properties, and sensory indexes of Smoke 73 grape juice were determined and analyzed. The results of the study showed that among the seven sterilization methods, total polyphenol content (TPC) in juice was significantly increased in all treatments except HHP. TPC was the highest in TP (3773.33 mg GAE/L). Total anthocyanin content (TAC) was increased except IR5, and TSN (1202.67 mg/L) had the highest TAC. In terms of color, TP (a* = 36.57, b* = 19.70, L* = 14.81, C* = 41.55, h° = 28.30, ΔE = 5.9) promotes the dissolution of anthocyanins because of high temperatures, which basically improves all the color indicators of grape juice and makes the color of grape juice more vivid. After HHP treatment, the color (ΔE = 1.72) and aroma indicators are closer to the grape juice itself. The Entropy weight-TOPSIS, CRITIC-Topsis, and PCA integrated quality evaluation models showed that all selected TP as the best integrated quality.

## 1. Introduction

With the rise of health trends, high-anthocyanin foods are becoming more and more valued by consumers for their wide range of health benefits. Yan 73 (*Vitis vinifera*) is a characteristic red grape variety cultivated in China and obtained by a cross between Muscat Hamburg (*Vitis vinifera*) and Alicante Bouschet (*Vitis vinifera*) in 1966 [1]. The skin and flesh of the grape contain a large amount of anthocyanins [2], and the total amount of anthocyanins in the grape skin can even reach 20–30 g/kg dry weight [3]. Grape has been shown to increase serum antioxidant capacity, alleviate atherosclerosis, prevent coronary heart disease [4], and attenuate oxidative stress caused by exercise [5], as well as possess anti-inflammatory, anticancer, and anti-aging [6,7] effects. In recent years, people have been paying increasing attention to disease prevention and treatment and healthy diets, and consumers pursue the nutrition, green, health, and convenience of diets so nutrition and health-oriented fruit and vegetable processing has attracted attention. In order to meet the consumer demand for nutritious and healthy food in the context of the new era, it is necessary to establish a key technology system for the efficient retention and steady-state processing of high-anthocyanin food active substances.

Sterilization is the key technical operation to ensure the stability of the juice and extend the shelf life of the juice, so it is very important to reduce the nutritional loss of grape juice in the sterilization process while maintaining the corresponding appearance of the color. Thermal pasteurization (TP) is a commonly used technique for sterilization, and the basic principle is to use high temperatures to inactivate enzymes and microorganisms as well as to degrade heat-sensitive compounds to prolong the shelf-life of food. However, high-temperature sterilization treatment may cause the degradation of heat-sensitive nutrients, affecting sensory and functional substances [8]. In recent years, with the continuous deepening of consumers’ pursuit of nutrition and health, non-thermal technology has emerged rapidly. It mainly includes ultrasonic sterilization (US), thermosonication (TS), TS combined antibacterial agent sterilization, irradiation (IR), high hydrostatic pressure (HHP), pulsed electric field (PEF), microfluidization (MF), and other technologies [9,10].

Currently, research on the sterilization of grape juice has mostly focused on exploring and improving one or two sterilization methods [11,12], such as exploring the efficacy of ultraviolet radiation as a substitute for SO_2_ in inactivating microorganisms in grape juice and wine [13], as well as the bactericidal effect of ultrasound combined with nisin on grape juice tested by Ma et al. [14]. The modification of the pulsed light (PL) sterilization system, such as the use of a spiral continuous circulation PL sterilization system, can improve the efficiency of PL treatment by increasing exposure in a limited space. The results of the study focused more on the effect of sterilization and less on its impact on the functional components and sensory properties of the juice, but it lacked a comprehensive comparison of multiple sterilization methods on grape juice. As a dyed grape variety rich in anthocyanins, Yan 73 (*Vitis vinifera*) has high nutritional value. So far, no researchers have explored the impact of sterilization treatment on the quality of fruit juice. At the same time, the impact and comparison of different sterilization methods on the functional components in fruit juice are still unclear.

Therefore, in this study, Yan 73 (*Vitis vinifera*) juice was sterilized by TP, TS, TS combined with nisin (TSN), TS combined with ε-Polylysine (TSε), IR, and HHP. Unpasteurized grape juice was used as a blank control (CK) to analyze the effects of different sterilization methods on the physicochemical components, sensory indexes, and functional properties of grape juice. We explore more excellent sterilization methods to provide a theoretical basis and an experimental basis for finding better grape juice sterilization technology.

## 2. Materials and Methods

### 2.1. Chemicals and Reagents

All standards and reagents, including 1,1-diphenyl-2-picrylhydrazyl (DPPH), catechins, and 6-hydroxy-2,5,7,8-tetramethylchromane-2-carboxylic acid (Trolox), were purchased from Sigma Aldrich (St. Louis, MO, USA); ε- Polysine and Nisin Z were purchased from Shanghai Yuanye Biotechnology Co., Ltd. (Shanghai, China).

### 2.2. Grape Juice Preparation

Yan 73 (*Vitis vinifera*), with a TSS of 18.1 ± 0.12 °Bx and titratable acid of 7.63 ± 0.22 g/kg from Zhangyu Ruina Winery, Xianyang, China, was selected for the experiment, and the grapes were planted in 2010. Based on the scheme explored by Wang et al. with minor adjustments [15], we washed the grapes, dried them, squeezed the juice, separated the juice from the must, and kept the skin residue. The skin residue was packaged in a bag that could be circulated with juice, and then pectinase 0.02% (*w*/*v*), cellulase 0.02% (*w*/*v*), and β-glucanase 0.04% (*w*/*v*) were added and enzymolized at room temperature for 6 h. It was then placed on a shaking table at 120 rpm, removed every 10 min, and stirred with a glass rod for 1 min (sealed with plastic wrap). After enzymolysis, the bag containing the circulatable peelings was squeezed, the peelings were fished out, and the skin residue was removed and placed in 4 °C cold storage waiting for sterilization.

### 2.3. Preparation of Bacteriostatic Suspension

Referring to the plan proposed by Sun et al. [16] and making slight adjustments, 0.05 g of Nisin Z with a potency ≥ 900 IU/mg was dissolved in 10 mL of pasteurized grape juice, filtered through a 0.22 µm filter to remove any microorganisms, and stored at 4 °C for later use.

### 2.4. Sterilization Treatment

**Figure 1:**
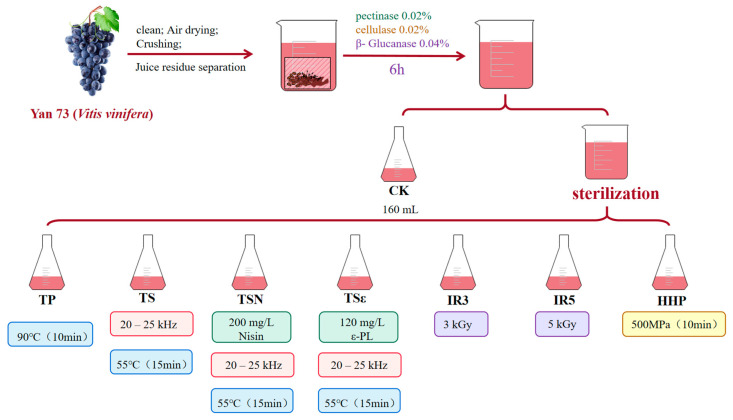
Treatment of seven sterilization methods.

As shown in Figure 1, grape juice was treated with TP, TS, TSN, TSε, IR3, IR5, and HHP, and the specific data for each treatment are shown in Table A1. The blended juice after enzymatic digestion was used as a blank control group (CK). All tubes were UV-sterilized for 2 h (pre-sterilized for 1 h on an ultra-clean bench), and the volume of grape juice for a single treatment was 160 mL, with at least three parallel experiments for each treatment.

TP was performed by treating the grape juice at 90 °C for 10 min using a thermostatic electric water bath [14]. For the TS, TSN, and TSε treatments, the prepared suspensions of Nisin Z and ε-PL were added to the grape juice to obtain the final concentrations of 200 mg/L [16] and 120 mg/L [17], respectively, and then uniformly treated at 55 °C for 15 min using an ATPIO-1000D built-in probe ultrasonography processor (Nanjing Xianou Ltd., Nanjing, China) with a frequency range of 20–25 kHz applied at a power of 700 W and connected to an XODC-0515-II thermostat (Nanjing Xianou Ltd., Nanjing, China). The IR3 and IR5 treatment groups relied on Yangling Hesheng Radiation Technology Co., Ltd. to treat a total of 10 bottles of grape juice (200 mL/bottle) with a total of 2 irradiation gradients of 3 kGy and 5 kGy [18,19]. For the HHP treatment, based on the experiments of Zhao et al. and with minor modifications [20], the juice was sealed in 20 cm × 32 cm homogenization bags (500 mL of grape juice, no more than 2/3 of the sample volume per bag) and processed at 500 MPa for 10 min using an HHP-700 hydrostatic pressurization unit (Bao Tou KeFa High Pressure Technology Co., Ltd., Inner Mongolia, China). The samples were treated at 500 MPa for 10 min.

### 2.5. Physicochemical Indicators

Conductivity was measured using a conductivity meter (Shanghai Leci Co., Ltd., Shanghai, China) and the results were expressed as mS/cm. The total soluble solids (TSS) were determined as °Bx using a TD-45 Digital Saccharimeter. Reducing Sugar (RS) and Titratable Acid (TA) were measured according to the national standard GB/T 15038-2006, and the results were expressed in g/L. The pH values were measured using a PHS-3E pH meter (Shanghai Leici Co., Ltd., Shanghai, China). Viscosity was measured by the NDJ-5S Rotational Viscometer (Shanghai Pingxuan Co., Ltd., Shanghai, China).

### 2.6. Functional Indicators

#### 2.6.1. Determination of Total Polyphenol Content (TPC), Total Anthocyanin Content (TAC), Total Flavonoids Content (TFC), Total Flavanol Content (TFOC), and Total Tannin Content (TTC)

TPC (expressed as mg gallic acid/mL sample), TFC (expressed as catechin equivalents), and TFOC (results expressed as (+)-catechin equivalents) were determined by the Folin–Ciocalteu colorimetric method, the aluminum chloride colorimetric method, and the p-DMACA-hydrochloric acid method. TAC was determined using the pH differential method, and the results were expressed as anthocyanin-3-glucoside (CGE, mg/L). TTC was determined spectrophotometrically with reference to the method of Varo et al. [21] and the results were expressed in g/L. Each treatment was repeated three times.

#### 2.6.2. Antioxidant Capacity Assay

Three methods were used to evaluate the antioxidant capacity of Yan73 grape juice: The 1,1-diphenyl-2-picryl-hydrazyl radical (DPPH) free radical scavenging assay, Ferrous reducing antioxidant capacity (FRAP), and trolox equivalent antioxidant capacity (ABTS) assay, with slight modifications following the method of Ma et al. [14]. The results are expressed as μmol Trolox/L.

### 2.7. Color Measurement

#### 2.7.1. CIELab

The CIELab parameters of grape juice were measured with the X-Rite ci7600 colorimeter (X-rite, Grand Rapids, MI, USA) in transmission mode, which directly obtained the values of L* (Luminance), a* (Green/Red), and b* (Blue/Yellow), while the chromaticity (C*), the hue (h°), and the total chromatic aberration (ΔE_ab_) were automatically calculated by the built-in software. Samples were measured in triplicate.

#### 2.7.2. Color Intensity (CI) and Tonality (T)

The samples were diluted 10-fold with deionized water and filtered through a 0.45 μm filter membrane, and the absorbance at 420, 520, 620 nm, and 700 nm was determined using a 10 mm light-range cuvette. CI was defined as the sum of ten times the absorbance at 620, 520, and 420 nm. Tonality was defined as the ratio of absorbance measured at 420 and 520 nm. The absorbance values, percentage yellow (Yellow%), percentage red (Red%), and percentage blue (Blue%) recorded at 420, 520, and 620 nm were also calculated according to the Glories method [22].

#### 2.7.3. Bathochromic Shift (Δλ_max_, nm) and Hyperchromic Effect (M)

The entire visible absorption spectra (400–700 nm) of all solutions were recorded using a UV-2800 UV-visible spectrophotometer (UNICO Co., Ltd., Shanghai, China) with intervals of 1 nm. Δλmax vs. M% was derived according to the method of Sun et al. [23].

#### 2.7.4. Polymeric Pigments Color (PPC)

Referring to the method of Varo et al. [21], 15 mg of Na_2_S_2_O_5_ was added to 5 mL of the grape juice sample, and it was allowed to stand at 25 °C for 45 min before being diluted 10 times. The absorbance at 520 nm was measured using a UV-2800 UV Vis spectrophotometer (UNESCO Co., Ltd., Shanghai, China), and the results were expressed in a.u.

### 2.8. Electronic Nose (E-Nose) Assay

The overall odor characteristics of grape juice were evaluated using PEN 3 Electronic Nose (Airsense Analytics, Schwerin, Germany), following the method of Lan et al. [24] with slight modifications. The specific parameters for electronic nose detection are as follows: Carrier gas speed of 300 mL/min, detection time of 60 s, cleaning time of 240 s, and at least 10 tests. The sensor response values of all samples reached equilibrium within 60 s, and the equilibrium stage response values were selected for analysis.

### 2.9. Comprehensive Quality of Sterilized BCJ Quantified by PCA, TOPSIS and GRA Models

The composite quality of Yan73 juice was assessed for composite quality based on the method of Bao et al. [25] with minor adjustments. The quality indicators can be categorized into four types according to their effectiveness: Maximum (the larger the value, the better the quality), minimum (the smaller the value, the better the quality), intermediate (the closer the value was to a certain value, the better the quality), and interval (the best quality of the sample when the value falls within a certain range). To calculate the composite quality score, the other three metrics need to be normalized and all converted to maximum before modeling. We constructed a matrix for evaluating the quality of Yan73 grape juice:

(1) $$X=\left[\begin{array}{c} x_{11}\;\;\;x_{12}\;\;\;\;\cdots \;\;\;x_{123}\\ x_{21}\;\;\;x_{22}\;\;\;\;\cdots \;\;\;x_{223}\\ \;\vdots \;\;\;\;\;\;\;\vdots \;\;\;\;\;\;x_{ij}\;\;\;\;\vdots \;\;\\ x_{81}\;\;\;x_{82}\;\;\;\;\ldots \;\;\;x_{823} \end{array}\right]$$

where *i* takes values in the range of 1–8, *j* takes values in the range of 1–23, and *x_ij_* represents the measured value of the *j*th indicator of the *i*th sample, as shown in Table A2.

The quality evaluation metrics screened above are normalized to the minimum value by Equation (2), where max is the largest value of the eight samples, *x_i_* is the respective value of each sample, and *y_i_* is the respective value of each sample after normalization. All the indexes were normalized to construct the Yan73 grape juice quality evaluation matrix Y after normalization.

(2) $$y_{i}=max-x_{i}\;Y=\left[\begin{array}{c} y_{11}\;\;\;y_{12}\;\;\;\;\cdots \;\;\;y_{123}\\ y_{21}\;\;\;y_{22}\;\;\;\;\cdots \;\;\;y_{223}\\ \;\vdots \;\;\;\;\;\;\;\vdots \;\;\;\;\;\;y_{ij}\;\;\;\;\vdots \;\;\\ y_{81}\;\;\;y_{82}\;\;\;\;\ldots \;\;\;y_{823} \end{array}\right]$$

The matrix Y was standardized according to Equation (3) to eliminate the effects caused by different index scales, and the standardized Yan73 grape juice quality evaluation matrix Z was obtained and subsequently analyzed.

(3) $$z_{ij}=\frac{y_{ij}}{\sum_{i=1}^{8}\sqrt{y_{ij}^{2}}}\;Z=\left[\begin{array}{c} z_{11}\;\;\;z_{12}\;\;\;\;\cdots \;\;\;z_{123}\\ z_{21}\;\;\;z_{22}\;\;\;\;\cdots \;\;\;z_{223}\\ \;\vdots \;\;\;\;\;\;\;\vdots \;\;\;\;\;\;z_{ij}\;\;\;\;\vdots \;\;\\ z_{81}\;\;\;z_{82}\;\;\;\;\ldots \;\;\;z_{823} \end{array}\right]$$

Based on the Z matrix, the Entropy weight-TOPSIS, CRITIC-Topsis, and PCA comprehensive quality evaluation model [25] was established, and the calculated weights of each index (Table A3) were used to calculate and rank the comprehensive scores of seven Yan73 grape juice samples.

### 2.10. Statistical Analysis

The organization of the data was performed in Excel 2020 (Microsoft Office, Redmond, WA, USA). Linear Discriminant analysis (LDA) and A one-way analysis of variance (ANOVA) were performed using SPSS 26 (IBM, Armonk, NY, USA) for statistical evaluation using Duncan’s multiple-range tests (*p* < 0.05). Bar charts were drawn using Graphpad Prism 8, and Origin 2023 performed heat map drawing, principal component analysis (PCA), and data analysis. Results are expressed as the mean of three replicates ± standard deviation (SD) for each treatment.

## 3. Results and Discussion

### 3.1. Effects of Different Sterilization Treatments on the Physicochemical Properties

**Figure 2:**
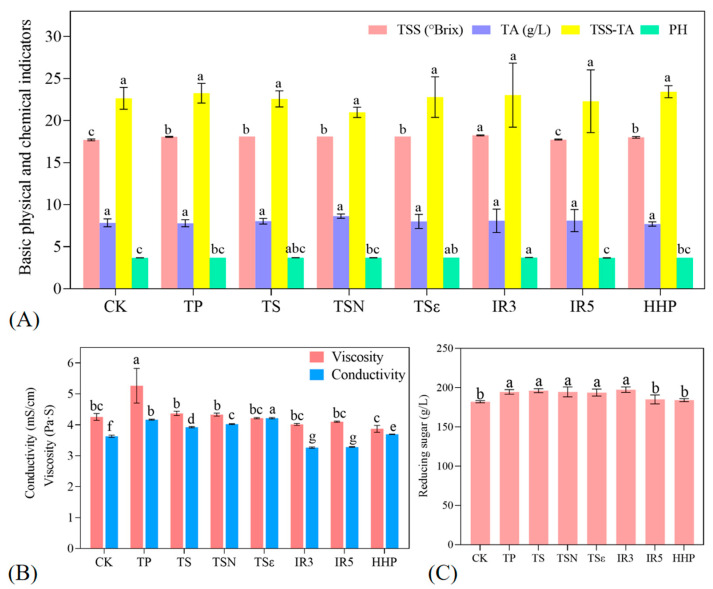
Effects of different sterilization treatments on the physicochemical properties of grape juice. (**A**) Total soluble solids (TSS), Titratable acid (TA), Sugar–acid ratio (TSS-TA), and pH; (**B**) viscosity and conductivity; (**C**) reducing sugar. Different lowercase letters in the figure indicate significant differences (*p* < 0.05).

The effect of different sterilization methods on the physicochemical properties of grape juice is shown in Figure 2. TTS and RS are important indicators reflecting the sweetness of grape juice. The TTS and RS of the CK group were 17.7 °Bx and 182.08 g/L, respectively. After sterilization treatment, the values increased, similar to the results of sterilization treatment of apple and orange juice [26]. Except for IR5, CK in the TTS treatment group was significantly increased (*p* < 0.05), the highest of which was IR3, which reached 18.23 °Bx. TP, TS, TSN, TSε, and IR3 in RS of eight treatment groups were significantly increased, while IR5 and HHP were not significantly different (*p* > 0.05). The highest was also IR3 (197.5 g/L). TA and pH, on the other hand, can respond to the acidity of the grape juice. The effect of the seven sterilization methods on the TA of the grape juice was not significant (*p* > 0.05), while the pH of the grape juice was significantly higher (*p* > 0.05) for the TSε and IR3 grape juices, with the highest being IR3. It can be seen that the size of the irradiation gradient affects the TTS, RS, and pH of grape juice differently. The sugar–acid ratios (TSS-TA) of the eight treatment groups were not significantly different (*p* > 0.05). In contrast to the results, it was found that there were no significant changes in TSS, pH, and TA of guava juice in TP and TS, likely because the energy level of ultrasound is unable to change the structures associated with the above features at the microscopic level [27]. TSS and pH also remained unchanged in the treatment of turbid pomegranate juice by TP and HHP [28].

Figure 2B shows that the viscosity of the CK grape juice was 4.25 Pa·s, the TP treatment showed a significant (*p* < 0.05) increase in must viscosity of 5.26 Pa·s, while the other fungicidal treatments showed no significant difference (*p* > 0.05) compared to CK. This was similarly demonstrated on HHP on carrot juice [25]. The increase in viscosity after TP treatment may be attributed to the “expansion” of juice particles and water infiltration between cellulose chains during the heating process [29] or to the complex polymerization of large molecules caused by thermal effects [14]. For conductivity, CK was 3.63 mS/cm. Except after IR, all other sterilization treatments significantly increased the conductivity of grape juice relative to CK (*p* < 0.05). The reason for this may be that the other treatments are the efflux of contents due to the disruption of the cellular structure [30], such as the release of minerals or vitamins from the tissues [31], resulting in an increase in the concentration of the amount of solids dissolved in the juice and an increase in the conductivity. The TSε treatment group had the highest conductivity of 4.22 mS/cm, likely due to the addition of the bacteriostatic agent ε-PL. The decrease in the conductivity of the juice after IR3 and IR5 treatments may be due to the ionization of the cells within the juice during irradiation and chemical interactions that impede charge transport.

### 3.2. Effects of Different Sterilization Treatments on the Polyphenol

#### 3.2.1. TPC and TAC

**Figure 3:**
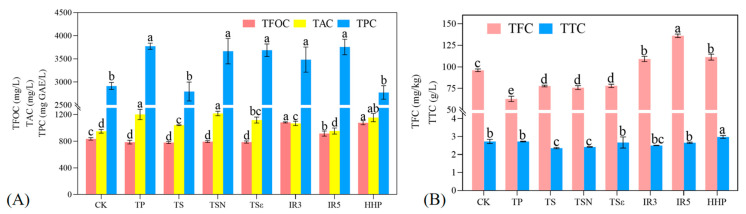
Effects of different sterilization treatments on the functional indicators of grape juice. (**A**) Total Flavanol Content (TFOC), Total Anthocyanin Content (TAC), and Total Polyphenol Content (TPC); (**B**) Total Flavonoid Content (TFC) and Total Tannin Content (TTC). Different lowercase letters in the figure indicate significant differences (*p* < 0.05).

The effects of seven sterilization methods on the functional characteristics of grape juice are shown in Figure 3. TPC and TAC in Figure 3A showed that TPC of TP (3773.33 mg GAE/L), TSN (3666.67 mg GAE/L), TSε (3686.67 mg GAE/L), IR3 (3483.33 mg GAE/L), and IR5 (3756.67 mg GAE/L) were significantly higher than that of CK (2913.33 mg GAE/L) (*p* < 0.05), and there was no statistical difference between TS and HHP and CK (*p* > 0.05). TPC in grape juice treated with TP was the highest, which increased by 29.52%. The reason may be that heat treatment induces the release of previously complexed or polymerized molecules and increases TPC by inactivating enzymes that catabolize TPC [32]. However, it was also found that TP and TS significantly reduced TPC in strawberry juice, and high temperatures can promote the reaction of polyphenols with oxygen due to the special hydroxyl structure of plant polyphenols [33]. TPC (increased by 28.95%) was the second highest in the IR5 treatment. This may be due to the activation of polyphenol biosynthesis pathways after IR treatment, such as the phenylpropanoid pathway, which is similar to the changes in polyphenol content in wine after irradiation treatment [34]. Some studies have also shown that the free radicals produced during irradiation may act as stress signals to induce the stress response of vegetables and increase the synthesis of antioxidants [19]. Studies have shown that IR significantly increases (*p* < 0.05) the TPC of fruit and vegetable juices such as carrot, ashitaba, and kale juices [18,19].

TAC was significantly higher (*p* < 0.05) in the treatments of other fungicidal modes except for IR5 (953.33 mg/L) and was highest in TSN (1202.67 mg/L) followed by TP (1202.67 mg/L) and HHP (1155 mg/L) compared to the CK group (949.66 mg/L). It is possible that the disruption of cell walls and vesicles by the sterilization treatment resulted in a faster rate of TAC release from Yan 73 grape juice than the degrading effect of the sterilization treatment on the original TAC. HHP treatment of wild goji berries under short-term low pressure (200 MPa) can increase the content of phenols and anthocyanins, mainly due to the improved permeability of the solvent through the structure [35]. Sterilization in most other juices such as blueberry, mulberry, and Hutai 8 grape juice reduces the total anthocyanin content to some extent [36].

#### 3.2.2. TFC and TFOC

As shown in Figure 3A,B, compared with the CK group, IR3, R5, and HHP in TFC and TFOC were significantly increased (*p* < 0.05), while TP, TS, TSN, and TSε were significantly decreased (*p* < 0.05). This may be due to the degradation of flavonoids by mechanical and cavitation effects due to ultrasound. Among them, IR5 had the highest TFC at 136.10 mg/kg, which increased by 41.76% compared to the CK (96.02 mg/kg). Next were HHP (111.32 mg/kg) and IR3 (109.18 mg/kg). The lowest content was found in TP with 62.77 mg/kg. In guava juice, TP resulted in a significant decrease (*p* < 0.05) in TFC, but TS was elevated by temperature treatments at 40 °C and 60 °C. This enhancement may be due to the combination of temperature and the cavitation effect of ultrasound disrupting the macromolecules and releasing bound forms of phenolic compounds [27]. Figure 3A shows that TFOC in the CK group was 835.00 mg/L. Among the seven sterilization methods, the highest TFOC was found in IR3 (1083.61 mg/L) and HHP (1078.06 mg/L), which were not significantly different (*p* > 0.05). The four fungicidal methods TP, TS, TSN, and TSε, did not show significant differences (*p* > 0.05).

#### 3.2.3. TCC

As shown in Figure 3B for TCC, the TCC of CK was 2.73 g/L. HHP (2.98 g/L) was significantly higher (*p* < 0.05) and TS (2.36 g/L) and TSN (2.42 g/L) were significantly lower (*p* < 0.05) than CK, while the other sterilization methods were not statistically different from the CK group (*p* > 0.05). The highest TCC was found in HHP (2.98 g/L), which increased by 9.16% compared to CK (2.73 g/L). TTC of grape juice treated with other sterilization methods decreased, with TS (2.36 g/L) showing the highest decrease at 15.68%. In studies of grape seed polyphenol extracts under thermal processing conditions, TCC usually shows a reduction. This may be due to the fact that the acidified butanol method does not react to the end units of oligomeric or polymeric tannins [37].

### 3.3. Effects of Different Sterilization Treatments on Antioxidant Activity

**Figure 4:**
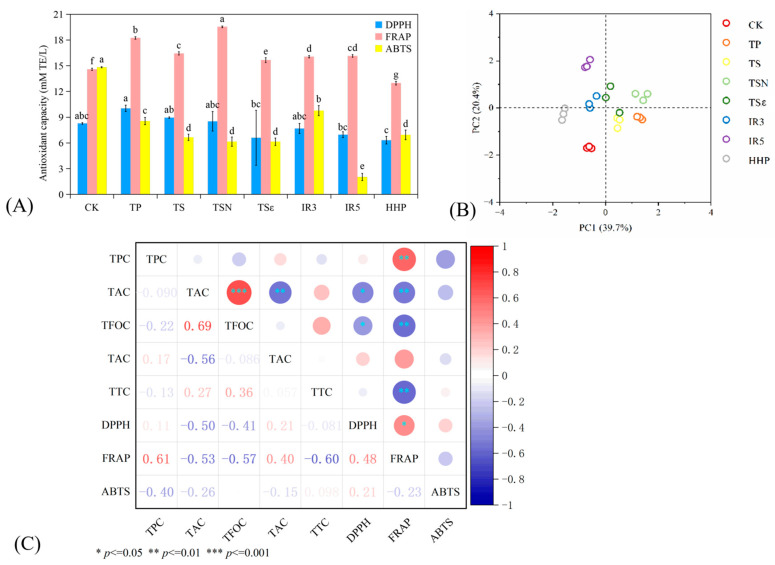
Effects of different sterilization treatments on the functional indicators of grape juice. (**A**) PPH scavenging activity, FRAP assays, and ABTS; (**B**) PCA score chart of phenols and antioxidant capacity; (**C**) correlation analysis between the phenols and antioxidant capacity. Different lowercase letters in the figure indicate significant differences (*p* < 0.05).

The antioxidant capacity of food depends on the composition of the active ingredients and the condition of the test system, as different antioxidants exert their antioxidant activity through different mechanisms, which cannot be fully assessed by a single method. Therefore, in this study, we evaluated the capacity of antioxidant activity of six sterilization methods on Yan 73 grapes using three methods: DPPH, FRAP, and ABTS, as shown in Figure 4A.

The antioxidant activities of DPPH, FRAP, and ABTS in the CK group were 8.28, 14.59, and 14.82 mmol TE/L, respectively. Compared with the CK group, the DPPH of the seven sterilization methods did not have a significant impact (*p* > 0.05). After TP (10.04 mmol TE/L) treatment, the increase rate of DPPH was 21.23%, followed by TS, 8.95 mmol TE/L (8.13%), and HHP (30.87%) showed the greatest decrease with 6.33 mmol TE/L. The DPPH value of TP in guava juice did not show a significant effect compared to CK, but TS was significantly higher (*p* < 0.05), which was also observed in blueberry juice. This increase may be attributed to an increase in polyphenols due to cavitation, which improves the accessibility and extraction of these essential ingredients [27]. The results of FRAP showed that the antioxidant capacity of HHP (12.97 mmol TE/L) was significantly reduced (*p* < 0.05) and significantly increased (*p* < 0.05) for all six other sterilization methods, with the largest FRAP value of 19.53 mmol TE/L for TSN. Seven sterilization treatments significantly reduced ABTS (*p* < 0.05), with the lowest decrease rate of 9.78 mmol TE/L in IR3 (34.01%) and the highest decrease rate of 2.03 mmol TE/L in IR5 (86.29%), indicating a significant relationship between ABTS and irradiation dose. Similar results were obtained in blueberry wine treated with ultrasound [16], and the scavenging activity of DPPH was not affected. Higher-efficacy ultrasound treatment resulted in a decrease in anti-ABTS, while FRAP increased. The increase in FRAP was positively correlated with the time of ultrasound treatment [38]. The principles of DPPH and ABTS are both free radical scavenging activities, and the coefficient of variation for ABTS was 48.07% among the eight groups of samples, compared to 16.04% for DPPH, suggesting that DPPH was less affected by the sterilization treatment, and therefore ABTS is likely to be more suitable for the assessment of antioxidant activity in the sterilization mode of Yan 73 grape juice samples.

PCA analysis of polyphenolics and antioxidant activity was performed for the sterilization methods of Yan 73 grape juice to determine which sterilization treatment was closer to the CK group in terms of juice characteristics. Figure 4B shows the PCA score plots of eight groups of samples with 39.7% variance contribution for PC1 and 20.4% for PC2, totaling 60.1%. In the PCA score plot, samples distributed in the same quadrant can be considered to have similar nutritional function performance, and it can be seen that both HHP and CK were in the third quadrant, and the combined performance of their nutrient content and antioxidant activity was similar. TSN and TSε were mainly distributed in the first quadrant, and it can be seen that the treatments with antibacterial agents were more similar. TP, TS, and TSε are distributed in the fourth quadrant, while IR3 and IR5 are mainly distributed in the second quadrant.

Figure 4C shows the correlation analysis between the phenols and antioxidant capacity. The red and blue colors in the right ruler represent positive and negative correlations, respectively. The number corresponds to a circle, and its absolute value is approximately close to 1. The darker the color, the larger the circle, and the stronger the positive/negative correlation between the two indicators. The closer the value is to 0, the opposite is true. It can be seen that DPPH is significantly negatively correlated with both TFC and TFOC (R_DPPH-TFC_ = −0.50, R_DPPH-TFOC_ = −0.41, *p* < 0.05). FRAP was significantly positively correlated with TPC (R_FRAP-TPC_ = 0.61, *p* < 0.01) and negatively correlated with TFC, TFOC, and TTC (R_FRAP-TFC_ = −0.53, R_FRAP-TFOC_ = −0.57, R_FRAP-TTC_ = −0.60, *p* < 0.01). This indicates that TPC contributes significantly to the antioxidant activity in Yan 73 grape juice. It has been shown that irradiated carrot juice has higher FRAP values than the non-irradiated control. Phenol content was significantly correlated with FRAP values (*p* < 0.01), but there was no correlation between the TPC and FRAP values of kale juice. This is due to the fact that ascorbic acid is extremely sensitive to irradiation, whereas the contribution of vitamin C to antioxidant activity may be much greater than that of irradiation-induced phenolic compounds. In addition, the combination of beta-carotene and other antioxidants in carrot juice provides synergistic protection against oxidation [19].

### 3.4. Effects of Different Sterilization Treatments on the Sensory Quality

#### 3.4.1. Color Analysis

**Figure 5:**
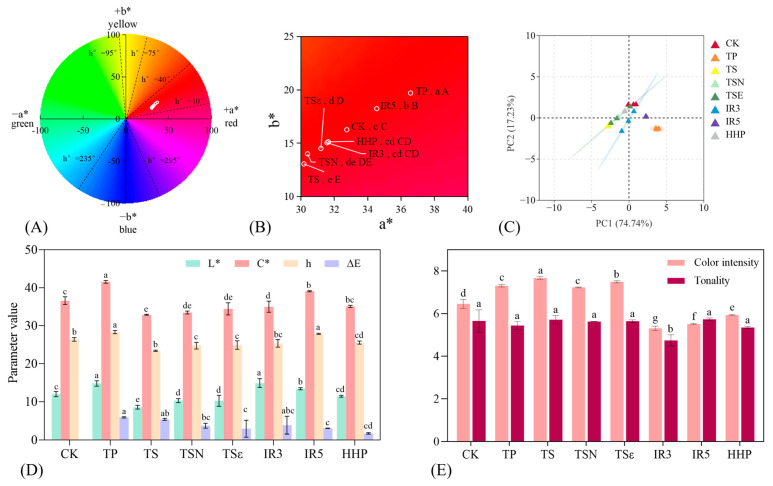
(**A**) Color characteristics of different sterilization treatments. (**B**) a*–b* value chromaticity distribution diagram; (**C**) PCA score diagram of different sterilization treatments; (**D**) changes in L* value, C* value, h value, and ΔE; (**E**) color intensity and tonality. Different lowercase letters in the figure indicate significant differences (*p* < 0.05).

Color is one of the important indicators when choosing drinks, which can not only reflect the quality of fruit but also make a certain contribution to the commercial value of fruit juice [39]. Many consumers expect flavor through the color of food, as an indicator of juice quality for selection [40]. The CIELAB color space is widely used to measure the overall color changes during juice processing and storage [41], as shown in Figure 5. Figure 5A,D show the color characteristics in the CIELAB space of Yan 73 grape juice treated with different sterilization methods. As shown in Figure 5A, a* (red-green) and b* (yellow-blue) indicate the color variation of the colors, and the a* values of the seven groups of samples were 30.17–36.57, while the b* values were 13.05–19.70. Among them, TP and IR5 were significantly increased (*p* < 0.05) in a* and b* compared to the CK group (a* = 32.75, b* = 16.26), suggesting that high temperature and high radiation levels may increase the redness and yellowness of Yan 73 grape juice. This is the same as the TP results for guava juice. Considering TSN, TSε, and TS compared to the CK group, a* and b* were significantly lower (*p* < 0.05), and ultrasonication may have decreased the redness and yellowness of the grape juice. However, during the sterilization of guava and spinach juices, thermal sonication resulted in an increase in the values of l* and b* and a decrease in the value of a*, and the cavitation fraction precipitated unstable and suspended particles, which formed additional colored substances [27]. Figure 5C shows the changes of L*, C*, h°, and ΔE in seven groups of samples, with L* ranging from 14.92–8.57 and C* values ranging from 32.88–41.55. Both TP and IR5 significantly increased the brightness and color saturation of grape juice (*p* < 0.05), and the overall color characteristics were improved. The h° was 23.39–28.30, within the red zone in the positive direction of a* and b*. ΔE > 2.8 values have been used to determine significant visual color differences in wines as perceived by the eye [42], and with the exception of the HHP treatment (ΔE = 1.72), all other methods of sterilization produced a significant change in the color of the juice. In the color analysis of pomegranate juice [43] and carrot juice [25], no visible color changes were observed during HHP treatment and with the naked eye, demonstrating better color protection effects. This is because ultra-high-pressure treatment does not damage covalent bonds, so low molecular compounds such as pigments in food are almost unaffected during the HHP process [25]. Figure 5B shows a plot of the PCA analysis scores for each parameter of the CIELAB color space, with a 74.74% contribution of variance for PC1 and 17.23% for PC2, for a total of 91.97%. It can explain the vast majority of the information in the original indicator variables. Among them, the HHP and CK groups are relatively close, indicating that this treatment has a small impact on the color characteristics of Yan73 grape juice. Relatively speaking, TP and TS have a greater impact on the overall color characteristics of grape juice. Liu et al.’s HHP and Ultra-high-temperature (UHT) treatments of watermelon juice showed that due to the combination of oxygen and high temperatures in the UHT-treated watermelon juice, the degradation of lycopene during storage was promoted, resulting in a* decreasing and b* remaining stable [44].

The color intensity (CI) and tonality (T) of grape juice are shown in Figure 5D. CI was involved in the formation of anthocyanin derivatives, and the color intensities of TP, TS, TSN, and TSε were significantly higher compared to the CK (*p* < 0.05). This may be due to the fact that higher temperatures can promote pigment dissolution and release, while ultrasound-induced cavitation interferes with pigment conversion and contributes to color [38]. Among them, TS had the highest CI of 7.67. Both IR and HHP treatments significantly reduced CI (*p* < 0.05). Except for IR3, other sterilization treatments did not significantly affect the T-value (*p* > 0.05).

**Figure 6:**
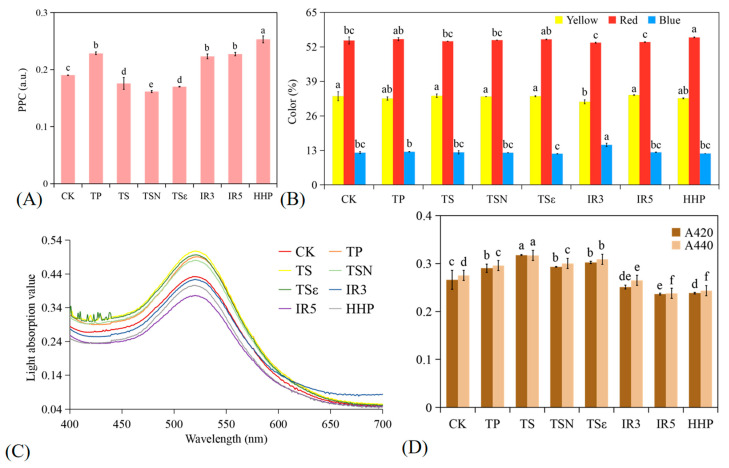
Color characteristics of different sterilization treatments. (**A**) Polymer color (PPC); (**B**) percentage of yellow, red, and blue colors; (**C**) light absorption value; (**D**) A420 and A440 of browning degree. Different lowercase letters in the figure indicate significant differences (*p* < 0.05).

The acylation of anthocyanins is called co-pigment deposition, which is driven by π-π stacking forces between aromatic rings, hydrogen bonds, and van der Waals interactions, resulting in changes in the optical properties of pigments. The configuration of co-pigment complexes relies on the formation of non-covalent complexes to protect anthocyanins from degradation, effectively improving their stability during processing and storage [23]. Δλ_max_ and M have been widely used as indicators of co-pigmentation. The specific data are shown in Table 1. As shown in Figure 6C, the absorbance variation curves of grape juice from 400–700 nm for 7 different treatments, TP, TSN, and TSε of λ_max_ increase compared to CK. The TP, TS, TSN, and TSε curves shifted upward with positive M%, while IR3, IR5, and HHP curves shifted downward, and the results were in good agreement with the changes in the CI size of grape juice. Heras-Roger et al. studied the color characteristics of red wines and showed that co-pigmentation mainly alters h° and decreases L, a*, and b* values, thus leading to purple and darker wines [45]. In the present study, except for TP, the results of treatments with increased M% of co-pigmentation rate are in line with that study, with purplish darkening of the grape juice color and decreased L, a*, and b* values.

Polymeric pigment color (PPC) is a pigment produced by the polymerization of anthocyanin and other phenolic substances, which stabilizes the color of the wine. Compared to the CK group (0.19 a.u.), PPC content in TP, IR3, IR5, and HHP was significantly higher (*p* < 0.05), and TS, TSN, and TSε were significantly lower (*p* < 0.05).

The absorbance values recorded at 420 nm, 520 nm, and 620 nm corresponded to the yellow percent (Yellow%), red percent (Red%), and blue percent (Blue%) of the grape juice, respectively. Among them, HHP Red% was significantly increased (*p* < 0.05), and IR3 caused a significant decrease (*p* < 0.05) in Yellow% and a significant increase (*p* < 0.05) in Blue% of the must. No significant changes were observed in the other treatment groups (*p* > 0.05).

The absorbance at 420 nm and 440 nm was considered the contribution of the brown hue and the degree of browning (BI) of the juice. Browning of juice is mainly due to the degradation of anthocyanins and the formation of brown compounds, corresponding to the presence of a brown hue caused by chemical and enzymatic browning reactions [46]. As shown in Figure 6D, the effects of different sterilization methods on the degree of BI of grape juice at both absorbance levels followed a similar trend. Relative to the CK group, the BI of TP, TS, TSN, and TSε was significantly higher (*p* < 0.05) and IR3, IR5, and HHP were significantly lower (*p* < 0.05). The results respond to the fact that heat treatment may deepen the browning of the juice and contribute to the non-enzymatic browning of the juice [43]. This is similar to the results of Gabriel et al. in heat treatment on grape seed polyphenol extracts [37]. The BI of strawberry juice was also significantly increased in TP and TS treatments. Γ irradiation induces a decrease in polyphenol oxidase activity, which is responsible for the browning of fruits and vegetables [19].

**Figure 7:**
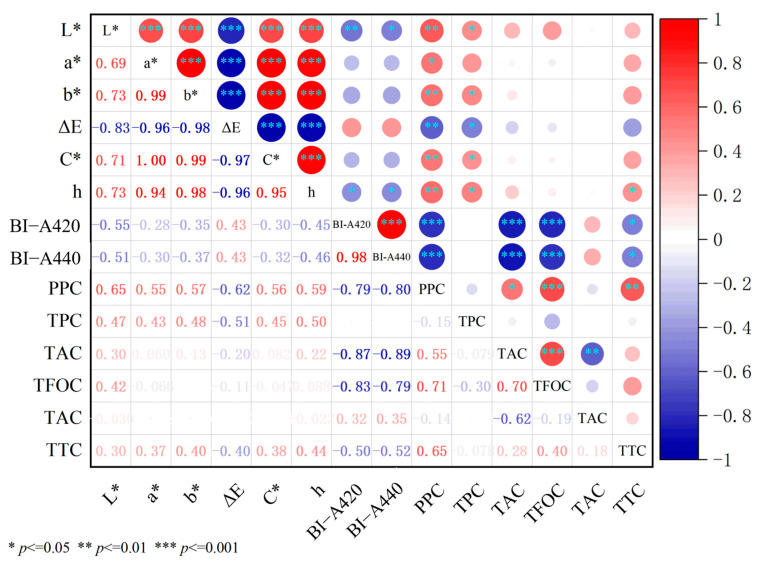
Correlation analysis between the color properties and antioxidant capacity.

The antioxidant active ingredients in the juice are closely related to the color characteristics, so correlation analysis between the color characteristics of the juice and the functional ingredients was performed, as shown in Figure 7. It can be seen that the BI of grape juice is significantly negatively correlated with TFC, TFOC, and TTC, while PPC is significantly positively correlated with them. TPC is significantly positively correlated with L*, b*, C*, and h. It can be seen that TPC has a significant contribution to the color of grape juice, which can increase the brightness and color saturation of Yan73 juice, making it more vibrant.

#### 3.4.2. E-Nose Analysis

**Figure 8:**
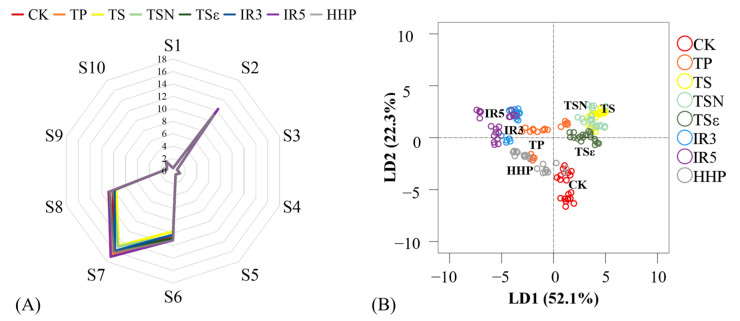
E-nose assay of grape juice after different sterilization treatments. (**A**) Radar chart of E-nose response data of grape juice after different sterilization treatments; (**B**) linear discriminant analysis (LDA) results of grape juice under different sterilization treatments.

As shown in Figure 8, a radar plot was made based on the response data of different sensors to the odor characteristics of grape juice (A). It can be seen that the overall trend of the response values of each sensor of the seven grape juice treatments is the same. The response values of S2 (sensitive to nitrogen oxides), S6 (sensitive to methyl compounds), S7 (sensitive to sulfides), and S8 (sensitive to alcohols, aldehydes, and ketones) sensors are relatively high, and their contribution to volatile gases in grape juice is relatively high. Among them, the response value of S2 sensor IR5 (12.32) was significantly higher than that of CK (9.91), and the response values of all other sensors were not significantly different. This indicates the increase or formation of some nitrogen oxides in IR5-treated grape juice. Furthermore, it has been shown that IR affects the aroma of fruit juices, such as the strong off-flavors observed in bitter melon juice [47].

The average stabilized signals of the ten E-nose sensors at 56–60 s were analyzed using the LDA linear discriminant method (Figure 8B), and the two discriminant functions explained 74.4% of the entire variance, with LD1 explaining 52.1% and LD2 22.3% of the variance. From the perspective of aggregation degree, for TS, TSN, and TSε, the aroma is more concentrated and has better consistency. Relatively speaking, the response value data of HHP are closer to CK, which to some extent indicates that its odor profile is closer to CK. HHP was closer to fresh juice compared to other treatments in the sterilization study of kiwifruit juice [20], and the same finding was found in mango juice [48]. HPP-treated Pennywort (*Centella asiatica* L.) juice samples retained more volatile compounds as shown by Arunee et al. [49]. Liu et al. on the other hand, found that esters, acids, alcohols, ketones, and alkanes were better retained in HHP-treated (400 MPa, 20 min) watermelon juice after 8 weeks of storage [44]. Therefore, HHP treatment may have a better effect on maintaining the flavor of the juice.

### 3.5. Comprehensive Quality of Sterilized Quantified by PCA, TOPSIS and GRA Models

The present study was conducted to evaluate the comprehensive quality of the juice on the basis of the index data of physicochemical properties, functional properties, and color characteristics of seven samples of Yan73 grape juice.

Since the physicochemical and color characteristics could not directly reflect the quality of the samples, and the differences in some indicators were not significant after different treatments, the samples within each treatment group were screened under the condition of significance (*p* < 0.05). Among the physicochemical characteristics, the increase in TSS, RS, and viscosity is mainly due to the release of sugar compounds, while the increase in conductivity is mainly due to the release of minerals and vitamins from the colloidal particles or cells of the fruit [50], which is therefore used as maximum. The lower the pH, the brighter the color of grape juice, so it was used as the minimum. The color profile was based on obtaining a fresher, steadier red Yan73 grape juice, and the larger the values of L*, a*, b*, C*, PPC, color intensity, λ_max_, and M (%) (maximum) and the smaller the values of BI-A420 and BI-A440 (minimum), the better the quality of the samples conformed to the standard, the results of which are shown in Table 2.

As shown in Table 2, the entropy weight-TOPSIS, CRITIC-Topsis, and PCA comprehensive quality evaluation models all ranked TP first in the comprehensive quality of Yan73 grape juice. The juice rankings for all other treatments were somewhat different. The main reason is that the weights of different indicators in each model account for different proportions. Entropy weight-TOPSIS and the PCA comprehensive quality evaluation are more inclined to the color characteristics in the comprehensive indicators, while CRITIC-Topsis of each part of the indicators accounts for a more balanced proportion. The second-place evaluation model is based on TSN processing, which mainly focuses on color characteristics. The average ranking of IR5 was eighth, which was due to its lowest ranking in the PCA model, which accounted for the largest proportion of functional indicators, indicating that IR5 treatment caused a greater loss of functional indicators of Yan73 grape juice. The average ranking of the CK group was seventh, which was similar to the ranking of the three models with great confidence. It can be seen that except for the IR5 treatment, the other six treatments improved the comprehensive quality of fruit juice. Unfortunately, due to the lack of clear criteria for selection, the indicators of electronic noses were not evaluated in a comprehensive ranking.

However, previous studies have shown that TP affects the aroma profile of juices, producing off-flavors that are cooked, sulfurous, or fermented [51]. This results in changes in the quality of the juice and affects consumer acceptance of the food. TP was found to increase the acidity of grape juice and orange juice, and γ-terpinene, which produces a bitter flavor, was evident in heat-treated samples of Pennywort (*Centella asiatica* L.) juice [49]. Artificial sensory tasting conducted by Zhao et al. on TP- and TSN-treated orange juice showed that TSN was significantly superior to TP due to the pronounced acidity in TP-treated orange juice, the lowest scores for odor attributes, and higher color scores for TSN [16]. Therefore, TSN may be more acceptable to consumers if consumer preferences and quality evaluation models are taken into account.

## 4. Conclusions

In this paper, the effects of TP, TS, TSN, TSε, IR3, IR5, and HHP processing methods on the overall quality of Yan 73 grape juice were systematically investigated. Based on the differences in the physicochemical properties, functional indicators, and sensory characteristics of juice, the research results showed that among the seven sterilization methods, TPC in Yan73 grape juice significantly increased except for HHP, and TP was the highest (3773.33 mg GAE/L). Moreover, TAC also increased except for IR5, and TSN (1202.67 mg/L) had the highest TAC. IR and HHP treatments significantly elevated TFC and TFOC, and HHP (2.98 g/L) significantly increased TTC. None of the seven sterilization methods had a significant effect on the DPPH radical scavenging capacity. The FRAP antioxidant capacity was significantly lower (*p* < 0.05) for HHP and significantly higher (*p* < 0.05) for all other six sterilization methods. All seven fungicidal treatments caused significant (*p* < 0.05) decreases in ABTS free radical scavenging capacity, with IR3 showing the lowest decrease (34.01%) and IR5 the highest (86.29%). In terms of color, HHP (ΔE = 1.72) did not produce color changes visible to the naked eye, which was closer to the color of the grape juice itself. TP, TS, TSN, and TSε treatments enhanced the PCC and BI of grape juice, and TS, TSN, and TSε caused purple darkening of grape juice color and reduction of L*, a*, and b* values. On the other hand, TP promoted the dissolution of anthocyanins due to high temperature, which basically improves all color indicators of grape juice, making the color of grape juice more vibrant. The entropy weight-TOPSIS, CRITIC-Topsis, and PCA comprehensive quality evaluation models were built, and each sterilization method except IR5 was ranked ahead of the CK. TP was the best comprehensive quality. However, if we consider consumer preferences and quality evaluation models comprehensively, TSN may be more acceptable to consumers. This experiment explored the effects of different sterilization methods on the physicochemical composition, sensory indexes, and functional properties of the Yan 73 (*Vitis vinifera*) juice to provide a theoretical basis and an experimental foundation for finding better sterilization technology for grape juice.

## Figures and Tables

**Table 1:** λmax and M% of seven sterilization methods.

	CK	TP	TS	TSN	TSε	IR3	IR5	HHP
λmax (nm)	520	522	520	521	521	520	520	520
M (%)	0	13.32	17.44	11.03	14.74	−1.97	−12.95	−6.04

**Table 2:** Comprehensive quality score and ranking of different sterilization treatments.

Sample	Entropy Weight-TOPSIS		CRITIC-Topsis		PCA		Average	
	Score	Rank	Score	Rank	Score	Rank	Score	Rank
CK	0.33	7	0.45	6	−4.69	7	−1.30	7
TP	0.67	1	0.65	1	8.84	1	3.39	1
TS	0.28	8	0.39	8	−0.78	5	−0.03	5
TSN	0.42	2	0.47	5	2.19	2	1.02	2
TSε	0.40	6	0.43	7	1.97	3	0.93	3
IR3	0.41	3	0.48	2	0.04	4	0.31	4
IR5	0.41	5	0.48	3	−5.05	8	−1.39	8
HHP	0.41	4	0.47	4	−2.52	6	−0.55	6

## Data Availability

The data presented in this study are available on request from the corresponding author.

## Appendix A

**Table A1:** Treatment measures for seven sterilization methods.

Sterilization Treatment	Abbreviation	Temperature	Ultrasonic Frequency	Bacteriostatic Agent	Irradiation Intensity	Pressure
Unsterilized grape juice	CK	-	-	-	-	-
Thermal pasteurization	TP	90 °C (10 min)	-	-	-	-
Thermosonication	TS	55 °C (15 min)	20–25 kHz	-	-	-
TS + Nisin	TSN	55 °C (15 min)	20–25 kHz	Nisin	-	-
TS + ε-Polylysine	TSε	55 °C (15 min)	20–25 kHz	ε-PL	-	-
Irradiation 3K	IR3	-	-	-	3 kGy	-
Irradiation 5K	IR5	-	-	-	5 kGy	-
High hydrostatic pressure	HHP	-	-	-	-	500 MPa (10 min)

Note: “-” indicates that no treatment has been applied.

**Table A2:** Quality evaluation matrix of seven sterilization methods.

	CK	TP	TS	TSN	TSε	IR3	IR5	HHP
L*	12.01	14.81	8.57	10.29	10.26	14.92	13.42	11.44
a*	32.75	36.57	30.17	30.39	31.20	31.56	34.54	31.64
b*	16.26	19.70	13.05	14.00	14.50	15.04	18.24	15.12
C*	36.56	41.55	32.88	33.46	34.41	34.98	39.06	35.07
BI-A420	0.27	0.29	0.32	0.29	0.30	0.25	0.24	0.24
BI-A440	0.28	0.30	0.32	0.30	0.31	0.26	0.24	0.24
PPC	0.19	0.23	0.18	0.16	0.17	0.22	0.23	0.25
Color intensity	6.45	7.31	7.67	7.23	7.50	5.31	5.50	5.93
λmax	520.00	522.00	520.00	521.00	521.00	520.00	520.00	520.00
M (%)	0.00	13.32	17.44	11.03	14.74	−1.97	−12.95	−6.04
TPC	2913.33	3773.33	2793.33	3666.67	3686.67	3483.33	3756.67	2773.33
TFC	96.02	62.77	77.73	75.85	77.98	109.18	136.10	111.32
TFOC	835.00	786.39	780.83	794.72	787.78	1083.61	915.56	1078.06
TAC	949.67	1202.67	1041.33	1217.33	1118.33	1067.00	953.33	1155.00
TTC	2726.80	2724.20	2357.60	2422.00	2667.50	2505.80	2655.90	2975.50
DPPH	8.28	10.04	8.95	8.53	6.60	7.70	6.97	6.33
FRAP	14.59	18.24	16.44	19.53	15.65	16.06	16.15	12.97
ABTS	14.82	8.56	6.66	6.16	6.14	9.78	2.03	6.95
TSS	17.70	18.07	18.10	18.10	18.10	18.23	17.73	18.00
pH	3.67	3.68	3.69	3.68	3.69	3.70	3.67	3.68
Viscosity	4.25	5.26	4.37	4.33	4.22	4.01	4.10	3.87
Conductivity	3.63	4.17	3.92	4.02	4.22	3.26	3.28	3.69
Reducing sugar	182.08	194.58	196.25	194.58	193.75	197.50	185.00	184.17

**Table A3:** Weight of quality indexes under different models.

Qualities Indexes	Entropy Weight	CRITIC	PCA			
			**PC1**	**PC2**	**PC3**	**PC4**
L*	3.04%	3.73%	−0.14	0.28	0.28	−0.02
a*	5.52%	3.52%	−0.08	0.40	−0.02	−0.02
b*	3.98%	3.55%	−0.10	0.40	−0.01	−0.04
C*	4.93%	3.53%	−0.09	0.40	−0.02	−0.03
BI-A420	3.38%	4.80%	−0.31	0.04	0.10	0.03
BI-A440	4.15%	4.83%	−0.31	0.04	0.07	0.01
PPC	4.16%	4.16%	−0.23	0.16	0.16	0.28
Color intensity	3.70%	4.90%	0.29	0.01	−0.21	0.09
λmax	12.39%	3.63%	0.20	0.29	0.08	0.16
M (%)	2.92%	4.85%	0.31	−0.02	−0.04	0.10
TPC	4.53%	4.00%	0.06	0.23	0.28	−0.27
TFC	3.82%	5.41%	−0.30	−0.07	0.06	−0.20
TFOC	8.79%	5.06%	−0.24	−0.10	0.29	0.22
TAC	4.13%	4.13%	0.17	0.08	0.23	0.40
TTC	3.48%	4.40%	−0.18	0.13	−0.14	0.49
DPPH	4.10%	4.12%	0.19	0.20	−0.04	−0.12
FRAP	2.68%	4.26%	0.21	0.15	0.17	−0.34
ABTS	2.73%	4.89%	0.01	0.00	−0.17	0.21
TSS	3.13%	4.78%	0.18	−0.11	0.43	0.22
pH	2.37%	4.59%	−0.11	0.20	−0.42	−0.06
Viscosity	4.63%	3.64%	0.19	0.33	−0.04	0.03
Conductivity	3.93%	4.54%	0.27	0.07	−0.14	0.28
Reducing sugar	3.51%	4.71%	0.23	−0.04	0.38	−0.07
